# Proteomic Profiles in Acute Respiratory Distress Syndrome Differentiates Survivors from Non-Survivors

**DOI:** 10.1371/journal.pone.0109713

**Published:** 2014-10-07

**Authors:** Maneesh Bhargava, Trisha L. Becker, Kevin J. Viken, Pratik D. Jagtap, Sanjoy Dey, Michael S. Steinbach, Baolin Wu, Vipin Kumar, Peter B. Bitterman, David H. Ingbar, Christine H. Wendt

**Affiliations:** 1 Department of Medicine, University of Minnesota, Minneapolis, Minnesota, United States of America; 2 Minnesota Supercomputer Institute, University of Minnesota, Minneapolis, Minnesota, United States of America; 3 Department of Computer Science and Engineering, University of Minnesota, Minneapolis, Minnesota, United States of America; 4 School of Public Health, University of Minnesota, Minneapolis, Minnesota, United States of America; 5 Minneapolis VA Medical Center, University of Minnesota, Minneapolis, Minnesota, United States of America; The Hospital for Sick Children and The University of Toronto, Canada

## Abstract

Acute Respiratory Distress Syndrome (ARDS) continues to have a high mortality. Currently, there are no biomarkers that provide reliable prognostic information to guide clinical management or stratify risk among clinical trial participants. The objective of this study was to probe the bronchoalveolar lavage fluid (BALF) proteome to identify proteins that differentiate survivors from non-survivors of ARDS. Patients were divided into early-phase (1 to 7 days) and late-phase (8 to 35 days) groups based on time after initiation of mechanical ventilation for ARDS (Day 1). Isobaric tags for absolute and relative quantitation (iTRAQ) with LC MS/MS was performed on pooled BALF enriched for medium and low abundance proteins from early-phase survivors (n = 7), early-phase non-survivors (n = 8), and late-phase survivors (n = 7). Of the 724 proteins identified at a global false discovery rate of 1%, quantitative information was available for 499. In early-phase ARDS, proteins more abundant in survivors mapped to ontologies indicating a coordinated compensatory response to injury and stress. These included coagulation and fibrinolysis; immune system activation; and cation and iron homeostasis. Proteins more abundant in early-phase non-survivors participate in carbohydrate catabolism and collagen synthesis, with no activation of compensatory responses. The compensatory immune activation and ion homeostatic response seen in early-phase survivors transitioned to cell migration and actin filament based processes in late-phase survivors, revealing dynamic changes in the BALF proteome as the lung heals. Early phase proteins differentiating survivors from non-survivors are candidate biomarkers for predicting survival in ARDS.

## Introduction

Acute Respiratory Distress Syndrome (ARDS) is characterized by the abrupt onset of tachypnea, hypoxia, and loss of lung compliance in response to infectious or inflammatory triggers [1]. Extensive research has improved our understanding of ARDS pathophysiology [2], epidemiology [3], [4], treatment options [5]–[7], and outcomes [3], [8], yet ARDS patients continue to have a high mortality rate. There is strong interest in identifying biomarkers to predict the development of ARDS in at-risk subjects [9]–[11], assist in diagnosis [12]–[15], and inform prognosis [13], [16]–[20]. Biomarkers enabling risk stratification would not only be useful in the clinical care setting, but also in clinical trials of new therapeutic interventions to phenotype clinical trial subjects and serve as surrogate endpoints.

Development of ARDS is associated with the activation of a large number of inflammatory mediators that damage the alveolar epithelium, endothelium, and basement membrane. Biomarkers based on the tissue of origin have been studied in both single center studies [11], [21], [22] and in NHLBI ARDS network cohorts [13], [16], [21]. Most studies have focused on investigating an individual biomarker in blood, bronchoalveolar lavage fluid (BALF), or urine. Markers of inflammation such as interleukin-1β [23], interleukin 6 [7], and soluble TNF receptor I and II [24] are associated with poor prognosis in ARDS. Markers of endothelial damage including ICAM-1 [7], [21], Angiopoeitin (Ang) [13], and Von Willibrand Factor (vWF) [25] correlate with higher mortality from ARDS. Poorer outcomes are also associated with higher plasma levels of SP-D (but not SP-A), a marker of type 2 alveolar epithelial cell damage [19], and receptor of advance glycation end products (RAGE), a marker of type 1 alveolar epithelial cell damage. Several other molecules, such as those involved in coagulation [26], damage to extracellular matrix [20], and oxidative stress (urine NO) [20], correlate with ARDS outcomes. A combination of biomarkers and clinical predictors was found to be superior to clinical predictors or biomarkers alone for predicting mortality in ARDS [27]. However, the identification of a single biomarker or a combination of biomarkers that could be widely used has remained elusive [28] due to lack of correlation between the biochemical marker, pathophysiological variables and clinical outcomes.

The primary aim of this study was to identify pathways of survival and stimulate new biomarker discovery by characterizing the BALF protein expression profile of ARDS survivors and non-survivors at different stages (early versus late) of disease progression. We analyzed medium and low abundant protein fractions in BALF samples by using contemporary high-resolution mass spectrometry (MS)-based proteomics techniques, along with quantitative labeling methodology. Our hypothesis was that patients who are able to survive ARDS would exhibit a distinct BALF protein profile during the early phase of mechanical ventilator support. Here, we show distinct differences in the BALF proteome between patients who survive ARDS from those who die. Moreover, the ontologies of differentially expressed proteins in late-phase survivors (cell migration and actin cytoskeleton organization) differ markedly from those in early-phase survivors, suggesting a critical role for these processes during lung repair. Enhancing these processes may provide new directions for therapy in ARDS.

## Methods

### Study population

The University of Minnesota Institutional Review Board Human Subjects Committee approved this study. Patients were recruited at the University of Minnesota Medical Center. Informed consent for study participation was obtained from either the patient or the patient's legal representative. The early-phase ARDS BALF samples were available from clinically indicated bronchoscopies with excess supernatant made available for these studies. The late-phase ARDS samples were excess supernatant BALF obtained from research bronchoscopies with consent from the patient or the surrogate. BAL (100 mls normal saline) was performed using standard protocol in either the right middle lobe or left upper lobe (lingual)

For this study, patients were grouped based on the timing of the bronchoscopy – conducted in either the early phase of ARDS (Day 1–7) or the late phase (Day 8–35), referenced to the initiation of mechanical ventilation (designated Day 1) – and the outcome at the time of discharge (non-survivor or survivor). We thus studied patients in the early phase who were grouped into survivors or non-survivors and late-phase survivors. Late phase non-survivors were not included in this study as not enough BALF was available to perform the protein expression profile. The APAHCHEE-II score was calculated to assess the severity of illness on the day of bronchoscopy for patients in early phase of ARDS as previously described [29].

### Sample preparation

BALF samples were processed as previously described [30] with some modifications. BALF containing equal amounts of protein from individual patients were pooled to collect a total of 4 mg protein for each group (early-phase survivors, early-phase non-survivors, and late-phase survivors). Pooled BALF was concentrated and desalted by centrifugation with an Amicon 3-MWCO spin filter (Millipore, catalog number UFC800396). To decrease the dynamic range, we enriched the medium and low abundance proteins by selectively immunodepleting the fourteen most abundant proteins in the concentrated samples on Seppro IgY 14 spin columns (Sigma-Aldrich, cat # SEP010). The Seppro IgY 14 spin columns deplete albumin, IgG, α_1_-antitrypsin, IgA, IgM, transferrin, haptoglobin, α_2_-macroglobulin, fibrinogen, complement C3, α_1_-acid glycoprotein, aplopoproteins A-1, A-II and B. Per the manufacturer's instructions, each sample was mixed in the dilution buffer to a final volume of 500 µl, loaded onto the immunoaffinity depletion column, and incubated for 15 minutes at room temperature. To prevent saturation of the column, 250 µg of protein was depleted at a time. The unbound medium and low abundance proteins were collected in the flow through. Pooled samples from each representative group was required to have adequate protein concentrations since immunodepletion results in >90% of the proteins being removed. An additional wash was performed with 0.5 ml of the dilution buffer. The depleted samples were then concentrated with an Amicon filter. A buffer exchange with 0.5 M triethylammonium bicarbonate (TEAB) was performed to remove TRIS, and the sample was concentrated with an Amicon filter. A Bradford protein assay was performed to quantify the enriched low abundant proteins.

### iTRAQ labeling and 2D LC-Orbitrap MS

Enriched medium and low abundance proteins (50 µg from early-phase survivors and non-survivors, 25 µg from late phase survivors) were digested by trypsin and labeled with iTRAQ reagent (AB Sciex, Foster City, CA) [30] for mass spectrometric analysis. The total peptide mixture was purified with an MCX Oasis cartridge (Waters, Milford, MA) before separation via two-dimensional liquid chromatography-mass spectrometry (2D LC-MS). LC and MS experimental details were previously reported [30]. Proteins were separated and concentrated offline in the 1^st^ dimension into 15 peptide-containing fractions, collected in 2-minute intervals on a C18 Gemini column (Phenomenex, Torrance, CA) at pH 10, and in the 2^nd^ dimension by a C18 reversed phase capillary LC with a nano LC system (Eksigent, Dublin, CA). Data-dependent acquisition of the 6 most intense peaks per LC fraction was performed on an Orbitrap Velos system, with HCD (higher energy collision induced dissociation) as the activation type for peptide tandem MS.

### Database search for protein identification and quantification

Each of the 15.RAW files generated from the Orbitrap Velos MS system were converted to mzML files by using msconvert, then converted to a ProteinPilot compatible Mascot Generic Format (MGF) with preselected iTRAQ reporter ions. The MGF files were searched against the Human UniProt database along with contaminant protein sequences (84,838 sequences in total; December 2012) using ProteinPilot version 4.5 and the following search parameters: Sample Type: iTRAQ 4-plex (peptide labeled); Cys-alkylation: MMTS; Instrument: Orbi MS, Orbi MS/MS; Run Quant; Use bias correction; Search focus on biological modifications and amino-acid substitutions; Thorough search and with a Detected Protein Threshold (Unused Protscore (Conf)): 10%. The ProteinPilot searches and subsequent generation of PSPEP (FDR) reports and protein and peptide-level summaries were generated within Galaxy-P [31]. Because MS data acquisition was performed on BALF samples after depletion of 14 high abundance plasma proteins, the high abundance proteins (or their fragment) were manually removed if they were present in the list of inferred proteins generated by ProteinPilot. Protein Summary with iTRAQ ratios (with early-phase survivors as the denominator for determining fold change) was processed through a workflow built within Galaxy-P so that it yielded UniProt accession numbers and gene names of differentially expressed proteins. The mass spectrometry proteomics data have been deposited to theProteomeXchange Consortium [32] via the PRIDE partner repository with the dataset identifier PXD001095.

### Statistical analysis

Differences in the clinical characteristics of the three participant groups were calculated by using ANOVA and, when appropriate, a post hoc Tukey test. For protein identification and quantification, multiple hypothesis correction was performed by controlling for false discovery rate (FDR), which measures the expected proportion of false positives among the statistically significant findings. The FDR cutoff was set at ≤1% (global) for protein identification in ProteinPilot. For quantification of protein abundance, each ratio (obtained by comparison of early-phase non-survivors to survivors or late-phase survivors to early- phase survivors) was compared to one; multiple hypothesis correction was performed by controlling the FDR set at ≤5% [33] and computing q-values with the *mafdr* routine in Matlab. Proteins with q-values less than 0.05 were retained for further analysis.

### Gene Ontology (GO) Enrichment Analysis

To gain insight into the biological significance of differentially expressed proteins, we used the Database for Annotation, Visualization, and Integrated Discovery (DAVID, http://david.abcc.ncifcrf.gov, search date 7/11/13) [34]. DAVID provides batch annotations to highlight the most relevant GO term associated with a gene (or protein) list. Of the three GO terms annotated to a gene (molecular function, biological process, and cell compartment), we limited the biological process annotation to differentially expressed proteins. Functional annotation clustering analysis in DAVID was used to identify the combinations of genes according to common biological function. DAVID generates an enrichment score for a group of genes indicating annotation term member associations in a given experiment. An enrichment score of 1.3 is equivalent to a non-log scale p-value of 0.05.

### Individual Protein Quantification

Levels for selected proteins were measured by ELISA with commercially available kits (BlueGene Life Science Advance (MUC5AC), R and D Systems, (MMP9 and SP-D), APC Biomaterials LLC (club cell secretory protein), Abcam (Kiniongen, Antithrombin III, Ceruplasmin, Plasminogen, Prothrombin), MyBioSource Inc (decay-accelerating factor, thoredoxin), AssyPro (Factor 12), Cloud-Clone Corp (Moesin), CusaBio (CD9), MBL International (S100A9) and CusaBio (Ezirin).

## Results

### Characteristics of study participants

We analyzed BALF samples from 22 unique ARDS patients (Table 1 and Table 2): 7 in the early-phase survivor group (mean ARDS day of sample collection  = 2.0±1.15 days), 8 in the early phase non-survivor group (mean ARDS day of sample collection  = 3.25±2.19 days), and 7 in the late-phase survivor group (mean ARDS day of sample collection  = 18.6±13.3 days). Although the mean age of patients in the early-phase non-survivor group was higher than in the other two groups, this difference was not statistically significant (ANOVA p-value  = 0.16). The three groups did not differ in the severity of gas exchange on the day of the bronchoscopy, APACHEE-II score, BALF leukocyte count, or BALF neutrophil count. The average time from onset of ARDS to death in the early phase non-survivor group was 19.9±14.5 days.

**Table 1:** Demographic and Clinical Characteristics of Subjects by Study Group.

Variable	Early-phase ARDS survivors (N = 7)	Early-phase ARDS non-survivors (N = 8)	Late phase ARDS survivors (N = 7)	p-value*
Age	42.29±11.43	58.13±20.49	47.86±10.07	0.16
Gender	M = 5, F = 2	M = 6, F = 2	M = 5, F = 2	
Immunocompromised/Immunecompetent	4/3	3/5	2/5	
APACHEE-II score	19.14±7.4	19.75±4.7		0.85
ARDS Day of BALF collection	2.0±1.15	3.25±2.19	18.6±13.3	<0.001#
PF Ratio on day of bronchoscopy	143.7±34.1	150±71.1	161.5±83.7	0.137
BALF WBC count (cells/µl)	496±342.6	364.4±408.7	451.1±471.5	0.75
BALF Neutrophils (%)	56.0±33.3	49.0±38.1	40.6±38.5	0.9327

*One-way analysis of variance (ANOVA) with Tukey post-test.

# Statistically significant difference between late-phase survivors and early-phase survivors (p<0.05) and between late-phase survivors and early-phase non-survivors (p<0.05), but no difference between early-phase survivors and early-phase non-survivors.

PF ratio- PaO_2_ to FiO_2_ ratio.

**Table 2:** Pulmonary history and clinical risk factors for ARDS in the study subjects.

Past Pulmonary History	Early-phase ARDS survivors (n = 7)	Early-phase ARDS Non-survivors (n = 8)	Late phase ARDS survivors (n = 7)
None	3	6	1
VTE	1	0	0
Smoker (prior or current)	1	0	3
Lung infection	2	0	0
COPD	0	0	1
NSCLC	0	2	1
Prior ARDS	0	0	1
**Risk factor for ARDS**			
Disseminated candidiasis	1	0	0
Sepsis	4	2	0
Pneumonia, not specified	0	1	
Pneumonia, gram negative	0	1	2
Pneumonia, gram positive	0	2	0
Pneumonia, fungal	0	1	0
Pneumonia, viral	0	0	1
Pneumonia, aspiration	2	0	3
Pancreatitis	0	1	0
Unknown	0	0	1

### Proteins identified by peptide spectral matching and database searching

The ProteinPilot PSPEP FDR Summary reported 20,601 spectra matched to 10,355 distinct peptides at ≤1% global FDR for a total of 792 inferred proteins (Table S1, Protein Pilot PSPEP summary and protein identified at 1% FDR tab). High abundance proteins or their fragments (Table S1, High abundance/contaminants tab) that were incompletely removed by the depletion column were removed manually from the protein list. Suspected contaminants or misidentified proteins such as trypsin, bovine albumin, and the reverse matches that occurred from use of the target decoy strategy for peptide identification were also manually removed. After exclusion of these proteins, the number of inferred proteins was 724 (Table S1, BALF proteome tab). These724 proteins were used as the background for GO enrichment analysis (i.e. the “universe of identified BALF proteome”). Of these 724 proteins, quantitative spectral data were available on 499 to allow determination of the bias corrected relative abundance in the two comparison groups for this study (Table S1, BALF with quantification tab). All but three proteins had at least two peptides used for identification. Bias factors for the two comparison groups were 2.8 for the early phase non-survivors to survivors and 0.64 for early phase survivor to late phase survivor group. Bias factors were used for normalization of the protein quantification within ProteinPilot.

### Proteins differentiating early-phase survivors and early-phase non-survivors

**Figure 1:**
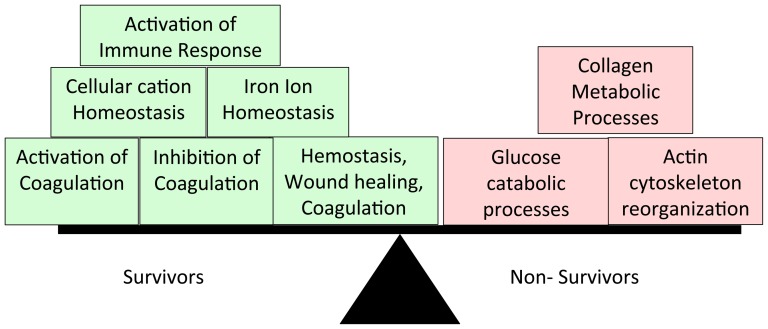
Biological processes represented by 165 proteins that are differentially expressed when early-phase non-survivors are compared to early-phase survivors. GO enrichment analysis was performed using the universe of identified BALF proteins as a background. A Functional Annotation Clustering tool was used to group related biological processes. Annotation clusters with an enrichment score >1.3 are shown. In the functional annotation-clustering tool, an enrichment score of 1.3 that corresponds to a non-log scale p-value of 0.05 was used as the cutoff for significance.

Controlling for an FDR of ≤5%, we identified 161 proteins that were differentially expressed in the BALF of early-phase survivors compared with early-phase non-survivors (Table S2, proteins with q-values ≤5%). Eighty-six of these proteins were more abundant in non-survivors (Table S2, high in non-survivors tab) and 75 were more abundant in survivors (Table S2, high in survivors tab). Gene ontology enrichment analysis demonstrated significant differences in the biological processes represented by these differentially expressed proteins (Figure 1). The differentially expressed proteins represented six ontologies in survivors (Table 3): three involved in coagulation (fibrinolysis and coagulation and wound healing), two representing cellular ion homeostasis, and one involved in immune activation. In contrast, differentially expressed proteins mapped to three ontologies in non-survivors (Table 4, Table S2, GO non-survivors tab). Non-survivors showed disruption of bioenergetics with evidence of carbohydrate catabolism and cellular damage as evidenced by disorganization of actin filament based processes. In addition, there was evidence of collagen biosynthesis in non-survivors early in ARDS.

**Table 3:** Early-phase ARDS Survivor Ontology Groups and Associated Proteins.

GO Biological Process	Official Gene Symbol	Protein Name	Fold change*
Positive Regulation of blood coagulation	AHSG	Alpha-2-HS-glycoprotein	0.21
	APOH	Apolipoprotein H	0.21
	HRG	Histidine-rich glycoprotein	0.34
	PLG	Plasminogen	0.35
	F12	Coagulation factor XII	0.36
	F2	Coagulation factor II	0.57
	SERPINF2	Serpin peptidase inhibitor, member 2	0.38
Negative regulation of blood coagulation	AHSG	Alpha-2-HS-glycoprotein	0.21
	APOH	Apolipoprotein H	0.21
	KNG1	Kininogen 1	0.24
	PLG	Plasminogen	0.35
	F12	Coagulation factor XII	0.36
	APOE	Apolipoprotein E	0.44
	ANXA5	Annexin A5	0.45
	F2	Coagulation factor II	0.57
	ANXA2	Annexin A2	0.81
Regulation of body fluid levels	SERPINC1	Antithrombin III	0.16
	APOH	Apolipoprotein H	0.21
	KNG1	Kininogen 1	0.24
	PLG	Plasminogen	0.35
	F12	Coagulation factor XII	0.36
	ANXA5	Annexin A5	0.45
	F2	Coagulation factor II	0.57
	ANXA2	Annexin A2	0.81
Cellular cation homeostasis	KNG1	Kininogen 1	0.24
	HPX	Hemopexin	0.30
	SFTPD	Surfactant protein D	0.38
	APOE	Apolipoprotein E	0.44
	F2	Coagulation factor II	0.57
	FTL	Ferritin, light polypeptide	0.62
	FTH1	Ferritin, heavy polypeptide 1	0.62
	55	Decay accelerating factor	0.78
	CP	Ceruloplasmin	0.84
Iron ion homeostasis	HPX	Hemopexin	0.30
	FTH1	Ferritin, heavy polypeptide 1	0.62
	FTL	Ferritin, light polypeptide	0.62
	CP	Ceruloplasmin	0.84
Positive regulation of immune response	C4BPA	Complement component 4 binding protein, alpha	0.09
	PLG	Plasminogen	0.35
	F12	Coagulation factor XII	0.36
	CFH	Complement factor H-related 2	0.45
	C1RL	Complement component 1r	0.56
	F2	Coagulation factor II	0.57
	CLU	Histone cluster 1,	0.65
	C5	Complement component 5	0.66
	KRT1	Keratin 1	0.67
	CD55	Decay accelerating factor	0.78
	C8A	Complement component 8, alpha	0.79
	C6	Complement component 6	0.80
	APOH	Apolipoprotein H	0.21

*Fold change is relative to survivors, therefore a fold change <1represents proteins more abundant in survivors.

**Table 4:** Early-phase ARDS Non-Survivor Ontology Groups and Associated Proteins.

GO Biological Process	Official Gene Symbol	Protein Name	Fold change
Actin filament-based process	TMSB4X	Thymosin-like 2	2.65
	EZR	Ezrin	2.15
	PFN1	Profilin 1	1.93
	VASP	Vasodilator-stimulated phosphoprotein	1.81
	CAP1	Adenylate cyclase-associated protein 1	1.58
	ARHGDIB	Rho GDP dissociation inhibitor (GDI) beta	1.52
	S100A9	S100 calcium binding protein A9	1.51
	FLNA	Filamin A, alpha	1.31
	MYH9	Non-muscle myosin, heavy chain 9	1.27
	STMN1	Stathmin 1	1.20
Glycolysis	GAPDHL6	Glyceraldehyde-3-phosphate dehydrogenase-like 6	2.01
	TXN	Thioredoxin	1.82
	PGK1	Phosphoglycerate kinase 1	1.73
	TPI1	Triosephosphate isomerase 1	1.63
	GPI	Glucose phosphate isomerase	1.63
	ENO1	Enolase 1, (alpha)	1.51
	PGAM1	Phosphoglycerate mutase 1	1.42
Collagen metabolic process	COL5A1	Type V collagen, alpha 1	2.33
	MUC5AC	Mucin 5AC	2.04
	COL3A1	Type III collagen alpha 1	1.99
	MMP9	Matrix metallopeptidase 9	1.84
	COL1A1	Type I collagen, alpha 1	1.75

### Changes in the proteome among late-phase survivors

**Figure 2:**
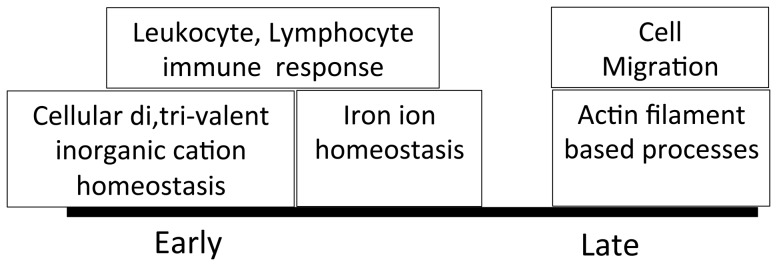
Biological processes represented by 175 proteins that are differentially expressed when late-phase survivors are compared to early-phase survivors. GO enrichment analysis was performed using the universe of identified BALF proteins as a background. A Functional Annotation Clustering tool was used to group related biological processes. Annotation clusters with an enrichment score >1.3 are shown. In the functional annotation-clustering tool, an enrichment score of 1.3 that corresponds to a non-log scale p-value of 0.05 was used as the cutoff for significance.

We identified 172 proteins (FDR at ≤5%) that demonstrated differential expression between early-phase and late-phase survivors of ARDS (Table S3, FDR q-values tab). Of these 172 proteins, 91 were more abundant in early-phase ARDS survivors (Table S3, high in early phase tab) and 81 were more abundant in late-phase ARDS survivors (Table S3, high in late phase tab). Gene ontology enrichment analysis employing the functional annotation-clustering tool in DAVID identified three ontology annotations that were over represented in early-phase survivors: lymphocyte and leukocyte immune response, cellular cation homeostasis, and iron ion homeostasis (Table S3, GO early phase tab). In contrast, proteins that were more abundant in late-phase survivors represented two clusters of ontologies involved in lung repair: cell migration and actin cytoskeleton organization (Figure 2 and Table S2, GO late phase tab).

### Changes in key proteins concentrations in individual samples

**Figure 3:**
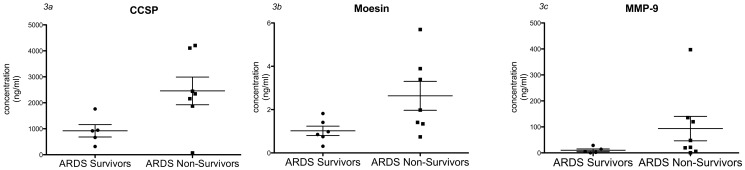
Protein levels of selected candidates. ELISA was performed to quantify CCSP, Moesin and MMP9. Levels of these proteins were higher in early phase non-survivors in comparison to survivor (p-value <0.05 t-test) for CCSP and <0.1 for Moesin.

As we used pooled samples for our proteomic studies, we measured protein concentration from individual BALF samples by ELISA. Similar to the MS data, the level of club cell secretory protein was significantly higher in early phase non-survivors when compared to early phase survivors (2458±1409 vs. 922±534 ng/mL, p-value  = 0.048, figure 3a). Moesin (1.02±0.52 vs. 2.63±1.76 ng/ml, p-value 0.055, Figure 3b) and MMP 9 (93.51±133.1 vs. 10±11.87 ng/mL, p-value  = 0.19, figure 3c) demonstrated a non-significant increase in early phase non-survivors compared to survivors. Although MUC5A was higher in survivors, it did not reach statistical significance (10.74±14.16 vs. 4.499±3.48, p-value  = 0.29 whereas SP-D was not different.

**Figure 4:**
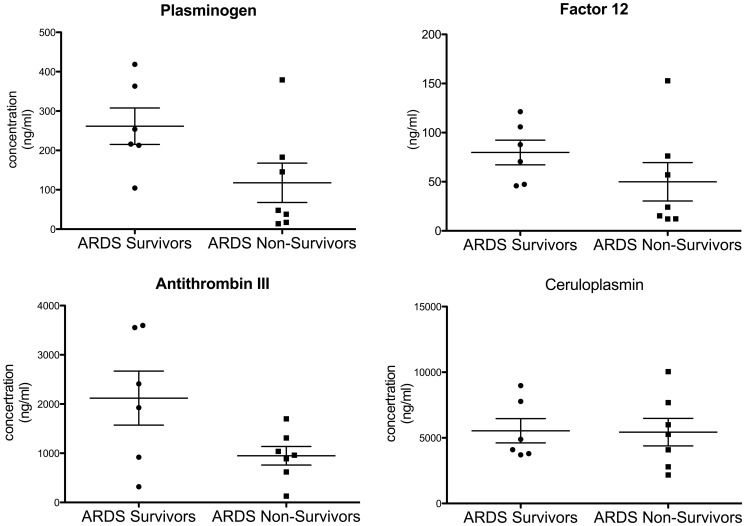
Protein levels of selected proteins that represent biological processes that are activated in early phase survivors (p-value plasminogen  = 0.06, antithrombin III = 0.054, factor 12 = 0.2 and ceruloplasmin  = 0.9).

**Figure 5:**
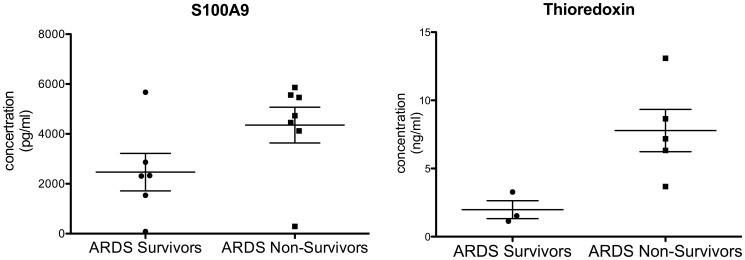
Protein levels of selected proteins that represented biological processes that are activated in early phase non-survivors. Thioredoxin was identified in only 3 survivors. (p-value <0.05 thioredoxin and <0.1 for S-100).

To develop a panel of candidate proteins that could discriminate early phase survivors from non-survivors, we measured BALF levels of several key proteins that were higher in survivors and participated in biological processes listed in Table 3. BALF levels of Plasminogen, Factor 12, Antithrombin and Cerulopasmin were consistent with our iTRAQ MS/MS findings (Figure 4). However, BALF levels of kininogen and Prothrombin (Factor 2) did not mirror the quantitative iTRAQ MS/MS data. We also measured levels of key proteins that participated in biological processes in early phase non-survivors listed in Table 4. S100A9 and Thioredoxin levels measured by ELISA mirrored the iTRAQ data (figure 5). Similar to the iTRAQ MS data, Ezrin level measured by ELISA in pooled BA was higher in non-survivors compared to survivors (2.45 ng/ml vs. 0.737 ng/ml.) We were not able to detect decay accelerating factor and CD9 by ELISA in our BALF samples.

## Discussion

In this study we achieved deep coverage of the BALF proteome through the use of high-resolution mass spectrometry based proteomics and an optimized sample preparation designed to enrich medium and low abundance protein fractions. This extent of proteome coverage has not been previously reported in human BALF from normal or diseased lungs [35]–[40]. The semi-quantitative techniques used in this study reveal dynamic changes in the distal airspace of patients with ARDS. Differences in the BALF protein expression profile are seen early in the course of ARDS in patients who die compared with those who live. Further, GO enrichment analysis demonstrates highly informative, biologically coherent differences in the ontologies represented by the differentially expressed proteins when early-phase survivors are compared to late-phase survivors or early-phase non-survivors.

The BALF protein expression profile for patients early in ARDS was different comparing survivors to non-survivors. Patients who died had evidence of aberrant lung repair early on in the disease process, as evidenced by approximately a two-fold differential expression of type I, III and V collagen, a signature of activated fibroblasts, and newly synthesized collagen deposition. This is in line with previous reports that higher levels of collagen I and III in ARDS [41] reflect matrix remodeling locally in the lung. Increased levels of BALF type III procollagen also correlated with fatal outcomes in ARDS [42]. In contrast, survivors early in the disease course demonstrated a more coordinated response that includes coagulation as the focal point, including plasminogen-mediated fibrinolysis. The balance of activation of coagulation and fibrinolysis is an important determination of the extent of fibrin deposition. In previous studies, tissue factor mediated pro-coagulant [43], protein C mediated anti-coagulant [44], and plasminogen mediated fibrinolysis pathways appeared to be important in ARDS [45]. Up-regulation of plasminogen activator inhibitor in BALF suggests a shift from a pro-fibrinolytic to an anti-fibrinolytic phenotype being associated with poorer outcomes [46]. In our study, a similar pattern of active collagen deposition suggesting an anti-fibrinolytic milieu even in the exudative phase in ARDS non-survivors is seen, while ARDS survivors have a more prominent fibrinolytic milieu. This indicates a profound difference in response to alveolar injury in survivors compared to non-survivors.

In addition to abnormal repair, non-survivors also have evidence of increased catabolism and cellular disruptions. In contrast, survivors demonstrate a coordinated activation of cation and iron homeostasis. Prior studies have shown the importance of iron in the development of ARDS [47], [48]. Cell and tissue damage resulting from inflammatory/oxidative stress can ultimately be a consequence of disruption of normal iron metabolism. Patients with ARDS have increased concentrations of heme and non-heme iron that could lead to generation of oxidative stress and resultant lung damage [49]. Polymorphisms in ferritin light chain and heme-oxygenase have also been associated with increased susceptibility to ARDS [50]. In survivors, higher levels of several proteins involved in iron regulation such as ferritin heavy and light chain, hemopexin, and cerruloplasmin indicate better capacity to counteract the redox stress mediated by iron or other reactive oxygen species in the lungs.

In addition to giving insight into mechanisms of disease, differentially expressed proteins in the early phase of ARDS can be used to discern non-survivors from survivors for prognostication. The ideal biomarker would have biological significance related to lung injury and repair. Alternatively, a panel of proteins that represent the divergent biological processes in the two groups could be selected for testing in a separate cohort of well phenotyped patients. As the proteomic platform that we used only provides relative quantitation, complementary studies using multiplex ELISA or multiple reaction monitoring will be needed to measure absolute levels to select a limited number of proteins that could be further investigated. We used ELISA for measurement of protein levels for two main reasons. First, this provided validation that mass spectrometric measures of the protein amounts were accurate. The fold change for CCSP (sp|P11684|UTER_HUMAN, Table S2, high in non-survivor tab, row 25) was 6.2 fold in the mass spectrometric studies. In line with these findings, the mean levels measured with ELISA demonstrated a >4.5 fold higher abundance in the protein level. Similarly, other proteins produced in the lung, Moesin, MMP9 and MUC5A, also demonstrated a trend toward higher levels in non-survivors by ELISA and mass spectrometry studies, whereas surfactant D did not demonstrate a significant change when measured in individual BALF samples. These proteins could represent epithelial damage and be candidate proteins to test in a larger cohort of well phenotyped subjects with ARDS.

CCSP is produced by small airway cells and has been implicated in regulating inflammatory responses in the lung. In patients with ventilator associated pneumonia, serum CCSP levels increased 2 days before the diagnosis of ARDS/ALI [51]. However, data regarding the utility of plasma CCSP levels is conflicting in small studies with one study demonstrating evidence in CCSP predicting mortality [52], while another study did not find any association of serum CCSP levels with mortality [53]. Our study suggests BALF CCSP levels alone or conjunction with other proteins could be marker of epithelial damage and could predict mortality in ARDS.

ERM (ezirin-radixin-moesin) proteins co-localize in cell matrix adhesion sites, filopodia, and membrane protrusions [54]. ERMs function by binding to and organizing the actin cytoskeleton [55] and in turn, stabilizing adherens junctions [56] and influencing cell migration [57], [58]. In adult wild-type mice, moesin expression is limited to the alveolar epithelium of the distal lung. Moesin-deficient mice develop normally [59] demonstrate decreased moesin in the distal alveolar wall and have airspace enlargement. In response to bleomycin, moesin- deficient mice had lower survival [60], more inflammation, extensive alveolar destruction, hemorrhage and pulmonary edema, increased lung permeability, and a higher total BALF cell count. In moesin-deficient mice, fibrotic response to bleomycin was both earlier and more severe. This supports involvement of moesin in injury-repair response in the lung.

Matrix metalloproteinase (MMP) are proteases that are involved degradation of extracellular matrix. Type IV collagen is specific to the basement membrane and MMP-9 is a type IV collagenase. In ARDS, BALF MMP-9 levels were high compared to controls and correlated with degree of collagen breakdown as determined by measuring collagen breakdown products (7S collagen) [61]. Early elevations of MMP-9 levels have also been found to be associated with prolonged duration of mechanical ventilation in pediatric ARDS patients [62]. In our study, although we did not compare the BALF MMP-9 level in ARDS with controls, higher BALF MMP-9 were seen in patients who died. Though speculative, this could be a marker of worse epithelial damage in non-survivors. In addition we also measured MUC5A levels in the BALF by ELISA. MUC5A is a member of the mucins, large glycoproteins that form a protective biofilm covering the respiratory epithelial lining. MUC5AC is secreted mostly by the surface epithelial goblet cells [63]. MUC5AC transcript levels increase in airway epithelial cells upon cyclic stretch, in mice with ventilator induced lung injury and in humans with ARDS [63]. Though the proteins levels of MUC5AC in our studies did not differentiate survivors and non-survivors, this may have resulted from our relatively small sample.

In this study we also compared the BALF protein expression profiles of early- and late- phase survivors. This comparison highlights the dynamic changes in the airspace milieu during repair. Chang and colleagues [36] characterized BALF in ARDS patients on day 1, 3, and 7; their results demonstrated striking differences between normal controls and ARDS patients on day 1, but less dramatic changes between days 1, 3 and 7. The changes seen in their studies reflected alteration in the innate immune and oxidant pathways at day 3 and possibly lung regeneration at day 7. Similar to that study, we found that activation of the innate immune system and cation homeostasis were over-represented by proteins in early-phase survivors compared to late-phase survivors. However, in our late-phase survivors – whose samples were obtained 18 days after the onset of ARDS, much later than in previous studies – the ontologies were drastically different and included cell migration and actin cytoskeleton organization. Since all of these patients survived, these findings suggest a critical role of these processes during lung repair. The proteins that are represented in these ontologies could be potential targets to stimulate repair mechanisms as potential molecular targets for therapy in ARDS.

We acknowledge the limitations of our study. We were limited by the amount of available BALF therefore are the protein characterization was performed on pooled BALF samples. Pooled samples are subject to influence by a minority of outliers within the pool. We were limited to a single run for the mass-spectrometry. However, we demonstrate changes in protein levels using ELISA studies that were performed on individual subject samples. Another limitation is the study design, i.e. binary outcome that can be influenced by a number of confounders not controlled in our study. Our approach was to enrich the medium and low abundance protein fractions by depleting high abundance proteins. We chose depletion over alternative method to avoid an unwieldy dynamic range with a subsequent limited depth of proteome coverage. In addition, many of the abundant proteins that were eliminated are found in the plasma, which can leak into the alveolar space during lung injury. Another limitation is our relatively small number of subjects. Despite the small sample size, our sample preparation optimization methods enabled us to successfully identify a number of differentially expressed proteins. Our findings provide a starting point for subsequent studies characterizing BALF in individual patients for biomarker identification in ARDS.

## Conclusion

This study illustrates a framework whereby protein profiling can be used to identify panels of proteins that parallel the pathophysiological changes occurring in ARDS. We demonstrate dynamic changes in BALF protein expression during the course of ARDS and also early divergence in the protein expression profile in ARDS. Differences in absolute levels of the proteins that represent divergent biological processes in survivors and non-survivors will facilitate identification of prognostic biomarkers in ARDS.

## Supporting Information

Table S1 **BALF proteins identified using iTRAQ MS/MS.**
(XLSX)

Table S2 **Differentially expressed proteins between early phase survivors and non survivors.**
(XLSX)

Table S3 **Differentially expressed proteins between early and late phase survivors.**
(XLSX)
